# 56600 Statin use and medical expenditure in patients with Parkinson’s disease

**DOI:** 10.1017/cts.2021.482

**Published:** 2021-03-30

**Authors:** Anthony J. Lo Piccolo, Alice C. Ceacareanu, Zachary A.P. Wintrob

**Affiliations:** 1 Hartwick College; 2 ROAKETIN Inc.

## Abstract

ABSTRACT IMPACT: Despite their clinical benefits reported in patients with Parkinson’s, statin use is not associated with cost savings. OBJECTIVES/GOALS: Statins have unique lipid-lowering, anti-inflammatory and anti-oxidant benefits. Their pleiotropic benefits were shown to decrease risk of occurrene and progression of Parkinson’s disease (PD). In this study we explored whether or not statin use reflects medical or prescription cost savings. METHODS/STUDY POPULATION: Records from the Medical Panels Expenditure Survey (MEPS) database made available by the Agency for Healthcare Research and Quality were used to identify all PD subjects (n=613). Demographics and PD ICD9/10 codes, 332/G20, were abstracted from the medical condition files for all the subjects (1996-2018). Prescribed cholesterol drugs were identified based on generic and brand names following a manual review to detect any misspellings. Total medical expenses and prescription expenses were abstracted for all identified PD subjects. Subject were surveyed for two consecutive years, thus expenses were assessed for each of the two surveyed years. Costs were adjusted for inflation and expressed in 2018 dollars. The relationship between cholesterol drug use, cost and age or gender was evaluated by Fisher’s exact test. RESULTS/ANTICIPATED RESULTS: Out of the 613 PD subjects identified, 421 received no cholesterol management, 15 received non-statins, 153 received a statin and 24 received a statin-based combo therapy. While the medical expenses in the general population receiving a statin are roughly three times higher than non-statin users, no significant cost difference was noticed between PD subjects receiving or not statins. However, after adjusting for age and gender, receiving statin vs. non-statin vs combo vs none was significantly associated with total expense (p=0.017) suggesting that cholesterol management decision may play a significant role. DISCUSSION/SIGNIFICANCE OF FINDINGS: Selection of specific cholesterol treatment may play a considerable role in the overall PD expenditure. Duration of statin treatment and type of statin are expected to play a role.

